# TMEM216 Deletion Causes Mislocalization of Cone Opsin and Rhodopsin and Photoreceptor Degeneration in Zebrafish

**DOI:** 10.1167/iovs.61.8.24

**Published:** 2020-07-20

**Authors:** Yu Liu, Shuqin Cao, Miao Yu, Huaiyu Hu

**Affiliations:** Center for Vision Research, Departments of Neuroscience and Physiology and of Ophthalmology and Visual Sciences, Upstate Medical University, Syracuse, New York, United States

**Keywords:** TMEM216, photoreceptor degeneration, tectonic complex, zebrafish, retina

## Abstract

**Purpose:**

Mutations in TMEM216, a ciliary transition zone tetraspan transmembrane protein, are linked to Joubert syndrome and Meckel syndrome. Photoreceptor degeneration is a prominent phenotype in Joubert syndrome. How TMEM216 contributes to photoreceptor health is poorly understood.

**Methods:**

We have generated *tmem216* knockout zebrafish by CRISPR genome editing. The impact of TMEM216 deletion on photoreceptors was evaluated by immunofluorescence staining and electron microscopy.

**Results:**

Homozygous *tmem216* knockout zebrafish died before 21 days after fertilization. Their retina exhibited reduced immunoreactivity to rod photoreceptor outer segment marker 4D2 and cone photoreceptor outer segment marker G protein subunit α transducin 2 (GNAT2). Terminal deoxynucleotidyl transferase dUTP nick-end labeling (TUNEL) revealed an increase in TUNEL-positive nuclei in the knockout retina, indicating photoreceptor degeneration. The *tmem216* mutation resulted in shortened photoreceptor ciliary axoneme, as revealed by acetylated α-tubulin immunostaining. Photoreceptors in knockout zebrafish exhibited mislocalization of outer segment proteins such as rhodopsin, GNAT2, and red opsin to the inner segment and cell bodies. Additionally, electron microscopy revealed that the mutant photoreceptors elaborated outer segment with abnormal disc morphology such as shortened discs and vesicles/vacuoles within the outer segment.

**Conclusions:**

Our results indicate that TMEM216 is essential for normal genesis of outer segment disc structures, transport of outer segment materials, and survival of photoreceptors in zebrafish. These *tmem21*6 knockout zebrafish will be useful in studying how transition zone proteins regulate photoreceptor outer segment formation and maintenance.

Joubert syndrome is a genetic disorder characterized by retinal dystrophy, cerebellar ataxia, oculomotor apraxia, hypotonia, neonatal breathing abnormalities, psychomotor delay, and renal disease. Neuroradiological examination of the brain on transaxial slices exhibit the diagnostic molar tooth sign resulting from hypoplasia/aplasia of the cerebellar vermis, thickened and reoriented superior cerebellar peduncles, and an abnormally large interpeduncular fossa.^1^^–^^3^ Defective primary ciliary signaling, resulting in developmental abnormalities, classifies it as a ciliopathy.^4^^–^^6^ Meckel syndrome (MKS), a more severe ciliopathy, characterized by early lethality with affected patients showing occipital encephalocele, kidney cysts and polydactyly.^7^^,^^8^ Both Joubert syndrome and MKS are genetically heterogeneous with mutations in multiple genes. Mutations in TMEM216,^9^^–^^13^ TMEM67,^14^^,^^15^ CEP290,^16^^–^^18^ RPGRIP1L,^19^ CC2D2A,^20^^–^^22^ TCTN1,^23^ and TCTN2^23^^,^^24^ have been linked to Joubert syndrome, as well as MKS in different individuals.

TMEM216 is a small protein with four hydrophobic putative transmembrane domains predicted to form two extracellular loops and one intracellular loop.^12^ TMEM216 knockdown resulted in a reduction in ciliogenesis.^12^^,^^25^ TMEM216 is a member of the transition zone tectonic complex.^12^^,^^26^ This protein-interacting complex consists of a group of Meckel and Joubert syndrome-related proteins including the secreted protein TCTN1, transmembrane proteins TCTN2, TCTN3, meckelin (TMEM67), and TMEM216, as well as intracellular proteins B9 domain–containing protein 1 (B9D1), CEP290, Meckel syndrome type 1 protein (MKS-1), and coiled-coil and C2 domain-containing protein 2A (CC2D2A).^26^ Similarly, an experiment aimed to identify proteins interacting with B9D1 found a similar set of proteins at the transition zone B9 complex including TCTN1, TCTN2, TMEM231, B9D1, MKS1, CC2D2A, and Jouberin.^27^ The tectonic/B9 complex is involved in the formation of cilia, regulates localization of ciliary membrane proteins such as Arl13b, and limits plasma membrane proteins in cilia.^26^^,^^27^ How these proteins contribute to photoreceptor survival is poorly understood because deleting some of these proteins in the mouse, such as cc2d2a,^28^ MKS1,^29^^,^^30^ TMEM67,^26^ and TCTN1 and 2,^26^ results in premature lethality before or shortly after birth, precluding detailed evaluation on photoreceptor survival. CEP290 mutant mice survive up to weaning, and photoreceptors show no outer segments growth with severely shortened inner segments.^31^

To evaluate the roles of TMEM216 in photoreceptor survival, we generated *tmem216* knockout zebrafish using CRISPR. Deletion of TMEM216 did not affect photoreceptor generation but resulted in their eventual degeneration. Photoreceptor degeneration is correlated with shortened cilia, mislocalization of outer segment proteins, abnormal organization of F-actin, and outer segment morphological defects.

## Materials and Methods

### Zebrafish Maintenance

AB/Tubingen strain zebrafish were purchased from Zebrafish International Resource Center (Eugene, OR, USA). They were housed in a recirculating water system (pH 6.6–7.4) at 26°C to 28.5°C with a daily cycle of 14 hours light:10 hours dark. Zebrafish were fed once a day with Gemma Micro (Skretting, Tooele, UT, USA). Protocols for all experimental handling of the animals were approved by the Upstate Medical University Institutional Animal Care and Use Committee and were in accordance to National Institute of Health guidelines and adhered to the ARVO Statement for the Use of Animals in Ophthalmic and Vision Research.

### In Situ Hybridization

Riboprobes for in situ hybridization were synthesized using reverse transcription–polymerase chain reaction (RT-PCR) to amplify a 483-base pair (bp) sequence in the zebrafish *tmem216* mRNA transcript. Primers were designed to insert T3 and T7 promoter sequences, as well as EcoRI and NotI cleavage sites on each end of the amplicon. The following RT-PCR primers were generated through Eurofins Genomics (Ebersberg, Germany): forward primer T3EcoR1TMEM216rtf6 (5′-AATTAACCCTCACTAAAGAATTCGTTTCATTTGAACGGCT-GGT-3′) and reverse primer T7Not1TMEM216rtr6 (5′-TAATACGACTCACTATAGGCGGCCGCGGTACTTGTCAGCT-TTATGTTTAATTG-3′). The RT-PCR product was analyzed by gel electrophoresis and was extracted and purified using the QIAquick Gel Extraction Kit (28706; Qiagen, Hilden, Germany). The purified RT-PCR product was subsequently amplified by polymerase chain reaction (PCR) and digested with EcoRI (R0101S; New England Biolabs, Ipswich, MA, USA) or NotI-HF (R3189L; New England Biolabs), according to manufacturer suggestions. In vitro transcription was performed using T3 RNA polymerase (EP0101; ThermoFisher, St. Louis, MO, USA) to synthesize the sense riboprobe and T7 RNA polymerase (EP0111; ThermoFisher) to synthesize the anti-sense riboprobe. The 10x DIG RNA labeling mix (11277073910; Sigma-Aldrich, St. Louis, MO, USA) was used in the in vitro transcription reactions to label each probe. Probes were purified using lithium chloride precipitation.

For in situ hybridization, zebrafish larvae were collected and fixed in 4% paraformaldehyde for 45 minutes, transferred to 20% sucrose for two hours and frozen in optimal cutting temperature (OCT) medium. Blocks were cryosectioned and collected on Fisherbrand Superfrost Plus Microscope Slides (Fisher Scientific, Waltham, MA, USA). Slides were washed in phosphate-buffered saline solution (PBS) for five minutes at room temperature, 100% methanol for 10 minutes at room temperature, and PBS and 0.1% Tween-20 three times for five minutes/wash. Slides were then treated with Proteinase K for 30 seconds and re-fixed in 0.2% glutaraldehyde/4% paraformaldehyde for 10 minutes. In situ hybridization was performed as described.^32^^,^^33^

### Generation of *tmem216* Knockout Zebrafish

We used clustered regularly interspaced short palindromic repeats (CRISPR)/Cas9 technology to generate *tmem216* knockout zebrafish. Three gRNAs targeting the coding region in exons 3 and 4 of zebrafish *tmem216* locus respectively, TCCTGTTTCATTTGAACGGCTGG and CCCACAAGATAATCTGATATTGG, were designed with E-CRISP online gRNA design tool (http://www.e-crisp.org/E-CRISP/designcrispr.html) and generated at Genscript (Piscataway, NJ, USA). A 25-µL microinjection mix consisting of 50 pmol gRNA and 50 pmol Cas9 protein (New England Biolabs) in TE buffer (10 mmol/L Tris-HCl pH 7.4 and 0.1 mmol/L ethylenediamine tetra-acetic acid) was prepared; 1 nL of this mix was then injected into zebrafish embryos at one- to two-cell stage. Screening of F0 for mutant zebrafish was carried out by PCR with forward primer CACTTTTGCAGGAAGACAACC and reverse primer GCATCGTCAAACACTGCTTC, followed by gel electrophoresis to identify the presence of amplicons of sizes bigger or smaller than the wildtype. F0 zebrafish that yielded shorter bands were used to cross with the wildtype zebrafish to obtain F1 zebrafish. F1 zebrafish were genotyped by PCR and PCR bands shorter than the wildtype (577bp) were purified and sequenced to identify mutations. This effort yielded two *tmem216* knockout lines (Fig. 2). The data in this study were collected from F2 and F3 generations.

### RT-PCR

Freshly laid eggs (pooled), 7 days post-fertilization (dpf) whole larva, 8 months post-fertilization (mpf) brain, 8-mpf muscle, and 8-mpf eyes were used to extract total RNA using RNeasy Plus Mini Kit (QIAGEN). RT-PCR was carried out using the S1000 Thermal Cycler (Bio-Rad) with the iTaq Universal SYBR Green One-Step Kit (Bio-Rad).

### Immunofluorescence Staining

Whole larvae without the posterior one-third were embedded in OCT medium and cryo-sectioned along the dorso-ventral plane. The posterior one-third of the larvae was used to extract genomic DNA for genotyping. Sections were collected on Fisherbrand Superfrost Plus Microscope Slides, fixed with 4% paraformaldehyde, and permeabilized with 0.1% Triton X-100 in phosphate buffer (PB). The sections were incubated with 3% bovine serum albumin (BSA) in 0.1 mol/L PB at room temperature for one hour in a humidified environment. The following primary antibodies were applied to the sections overnight at 4°C: anti-G protein subunit α transducin 2 (GNAT2; 1:400, PM075; MBL International, Woburn, MA, USA), anti-acetylated α-tubulin (1:100, Cat no. T6793; Sigma-Aldrich), anti-EYS (1:300, Novus Biological, NBP1-90038), 1D4 (1:1000, Abcam, AB5417), anti-CC2D2A (1:100, MBS 767569; MyBioSource, San Diego, CA, USA), anti-TMEM231 (1:100, PA5-42686; Invitrogen, Carlsbad, CA, USA) and anti-CHOP (1:300, 15204-1-AP; Proteintech, Rosemont, IL, USA). After washing with PB containing 0.1% Triton X-100, the slides were incubated with appropriate fluorescein isothiocyanate (FITC)-conjugated anti-rabbit IgG (1:300, 111-095-144; Jackson ImmunoResearch, West Grove, PA, USA) or rhodamine B isothiocyanate (RITC)-conjugated anti-mouse IgG (1:300, 115-025-146; Jackson ImmunoResearch) for 2 hours at room temperature in a humidified environment. After washing with PB containing 0.1% Triton X-100, the sections were counterstained with DAPI to visualize the nuclei. The sections were covered with VECTASHIELD mounting medium (H-1000; Vector Laboratories, Burlingame, CA, USA) by coverslips. To visualize fluorescence, a Zeiss Axioskop epifluorescence microscope (Carl Zeiss, Oberkochen, Germany) and a Zeiss LSM780 confocal microscope system were used. Epifluorescence images were captured with a mono 12-bit camera and QCapture Pro 6.0 (QImaging, Surrey, British Columbia, Canada). Terminal deoxynucleotidyl transferase dUTP nick end labeling (TUNEL) was carried out with ApopTag Fluorescein In Situ Apoptosis Detection Kit (Millipore, Burlington, MA, USA) according to the manufacturer's suggestions.

Fluorescence intensities of GNAT2, 1D4, and 4D2 were quantified using images taken with a ×40 objective lens on a Zeiss Axioskop epifluorescence microscope. Two images near the central plane of the retina were taken per animal. Images were imported to ImageJ software (National Institutes of Health) for quantification. Using ImageJ, the outer segment region of the retina was outlined, and the average fluorescence intensity for that region was calculated. Average background fluorescence was subtracted from the average fluorescence intensity to generate the reported adjusted average fluorescence intensity. Student's *t*-tests were performed to assess significance.

The length and number of acetylated α-tubulin axonemes were quantified using images taken with a Zeiss LSM780 confocal microscope with a ×40 oil objective lens. Two images near the central plane of the retina were taken per animal. Images were imported to ImageJ software to measure axoneme count and length. Student's *t*-tests were performed to assess significance.

### Western Blotting

Total protein was extracted from three wildtype and three *tmem216^snyR8Δ60^* homozygous knockout 7-dpf zebrafish larva heads using 40-µL radioimmuno precipitation assay buffer (50 mmol/L Tris pH 8.0, 150 mmol/L NaCl, 1.0% NP-40, 0.5% sodium deoxycholate, 0.1% sodium dodecyl sulfate (SDS)) with Halt Protease & Phosphatase Single-Use Inhibitor Cocktail (78442; Thermo Fisher Scientific, Waltham, MA, USA). Total protein along with Precision Plus Protein Standards (161-0374; Bio-Rad, Hercules, CA, USA) were separated on a 4% to 20% Mini-PROTEAN TGX Gel (456-1093; Bio-Rad) and transferred to polyvinylidene difluoride (PVDF) membranes. Membranes were washed three times in tris-buffered saline with 0.1% Tween-20 (TBST) (TBS, 0.1% Tween-20) for five minutes/wash and blocked with 3% BSA in TBST for one hour. After blocking, membranes were incubated with anti-GNAT2 (1:500, PM075; MBL International, Woburn, MA, USA) or anti-β-actin (1:1000, AS-55339; AnaSpec, Fremont, CA, USA) overnight at 4°C. After thorough washing with TBST, the membranes were incubated with horseradish peroxidase-conjugated goat anti-rabbit (1:2000) for two hours at room temperature and washed three times with TBST for five minutes/wash. Signal was developed using the SuperSignal West Pico Chemiluminescent Substrate (34580; Thermo Scientific).

Blot images were exported to ImageJ software for quantification. Each image was color-inverted, and the band was manually selected, in software, using the polygon selection tool. GNAT2 and β-actin band intensities were measured, and the ratio of GNAT2:β-actin intensity was calculated.

### Transmission Electron Microscopy (EM)

Zebrafish fry heads were fixed in 2% paraformaldehyde and 2% glutaraldehyde in phosphate buffer. Transmission EM was carried out as we previously described.^34^^,^^35^ For quantification of outer segment disruptions, photoreceptors with visible connecting cilia were selected for imaging spanning from the basal body to the outer segment at magnification × 40,000 or 60,000. Photoreceptors exhibiting shortened discs and vacuoles in the outer segment were counted. Fisher's exact test was performed to test for significance.

## Results

### Expression of *tmem216* in Zebrafish

**Figure 1.**
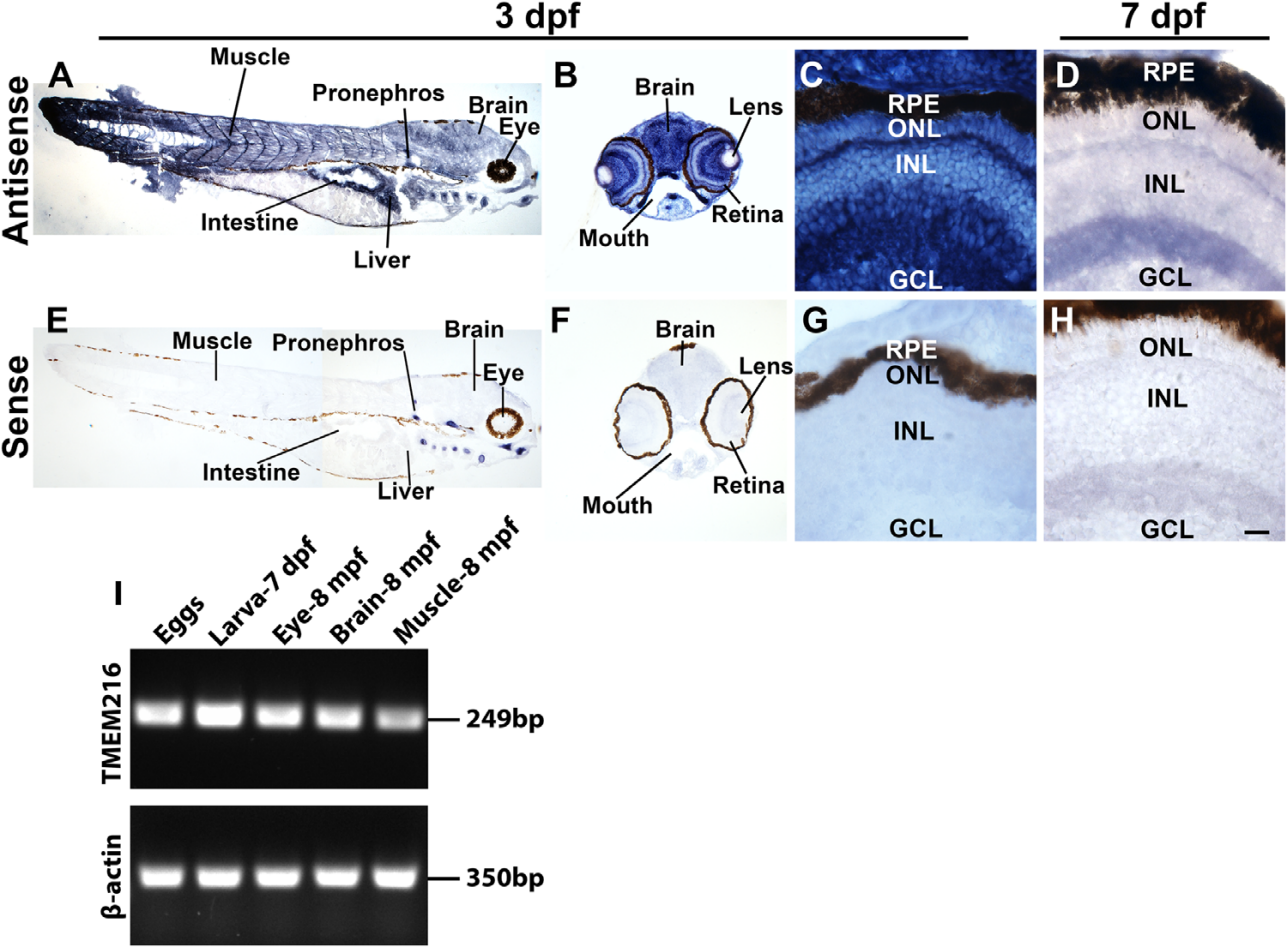
*tmem216* was widely expressed in zebrafish. In situ hybridization with *tmem216* sense and antisense probes was performed on cryosections of 3- and 7-dpf zebrafish larvae. (**A**) Antisense probe labeling of longitudinal sections of a 3-dpf larva. *tmem216* was widely expressed throughout the zebrafish, including the muscle, pronephros, brain, intestine, and liver. (**B, C**) Antisense probe labeling of 3-dpf zebrafish head. *tmem216* was widely expressed throughout the brain and neural retina. (**D**) Antisense probe labeling of 7-dpf zebrafish retina. *tmem216* expression persisted throughout the neural retina at 7-dpf. (**E–H**) Sense probe labeling of 3- and 7-dpf zebrafish cryosections. (**I**) RT-PCR of wildtype fish showing TMEM216 expression in newly fertilized eggs, 7-dpf larva, and 8-mpf eye, brain, and skeletal muscle. *Scale bar* in **H**: 200 µm for **A, B, E,** and **F**; 15.5 µm for **C, D, G,** and **H**.

To evaluate tissue expression of *tmem216*, we performed in situ hybridization with *tmem216* antisense probe (Figs. 1A–1D) using sense probe as a control (Figs. 1E–1H). At 3-dpf, in situ hybridization with antisense probe resulted in widely distributed signal in multiple organs including the eye, pronephros, brain, liver, intestine, and muscle (Figs. 1A and 1E). Within the retina, expression of *tmem216* was observed in all cell layers of the neural retina including the outer nuclear layer, inner nuclear layer, and the ganglion cell layer (Figs. 1B, 1C, 1F, and 1G). The *tmem216* expression was maintained in the 7-dpf zebrafish neural retina (Figs. 1D and 1H). RT-PCR showed that TMEM216 mRNA was detected in freshly laid eggs, 7-dpf larvae, and adult eye, brain, and skeletal muscle (Fig. 1I). These data suggest that *tmem216* is widely expressed in zebrafish.

### Generation of TMEM216-Deficient Zebrafish

**Figure 2.**
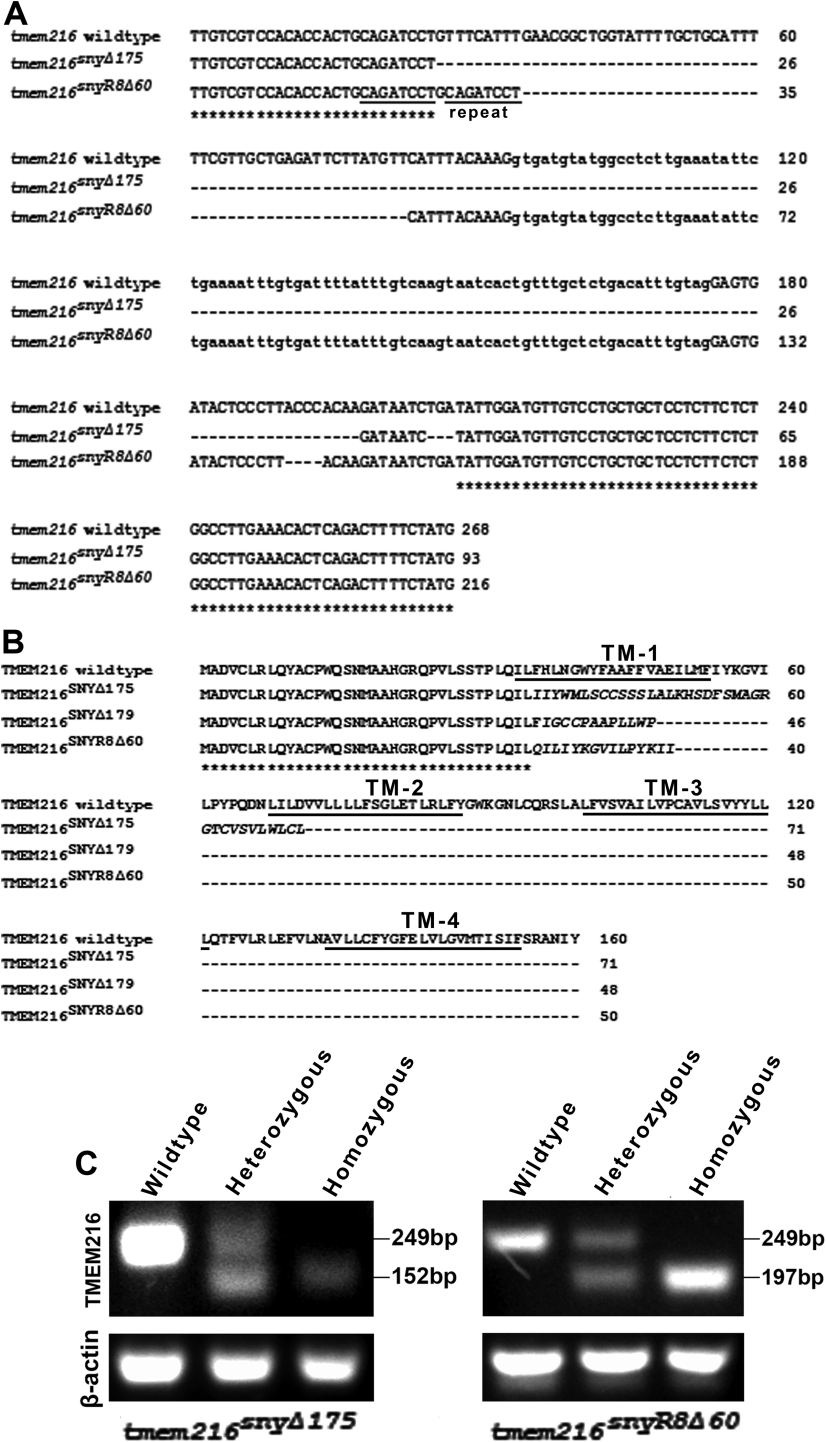
Two *tmem216* knockout zebrafish lines were generated by genome editing. Zebrafish knockout lines, *tmem216^snyΔ175^* and *tmem216^snyR8Δ60^*, were generated by CRISPR/Cas9 genome editing. (**A**) Alignment of mutant genomic sequences with wildtype sequence from exon 3 to exon 4. The *tmem216^snyΔ175^* line features two deletions of two stretches of DNA of 172 and three nucleotides. The *tmem216^snyR8Δ60^* line features an eight-nucleotide repeat and deletion of two stretches of DNA of 56 and four nucleotides. Both mutations were expected to result in frameshift. (**B**) Protein sequence alignment between wildtype TMEM216, and the expected TMEM216^SNYΔ175^ and TMEM216^SNYR8Δ60^ mutant proteins. Note both mutations result in the loss of all four transmembrane domains. (**C**) RT-PCR of wild-type, heterozygous, and homozygous zebrafish, showing that homozygous knockouts express only mutant mRNA.

To study the roles of TMEM216 in photoreceptor survival, we used CRISPR/Cas9 genome editing to generate *tmem216* knockout zebrafish. This effort yielded two knockout zebrafish lines, *tmem216^snyΔ175^*, and *tmem216^snyR8Δ60^* (Fig. 2A). The *tmem216^snyΔ175^* mutation harbors two deletions, a 172-bp deletion from exon 3 to exon 4 and a 3-bp deletion within exon 4. The *tmem216^snyR8Δ60^* mutation had an 8-bp duplication (CAGATCCT) within exon 3 and deletion of two fragments, a 56-bp deletion within exon 3, and a 4-bp deletion within exon 4. All of these mutations disrupted the reading frame and were expected to result in the loss of the majority of the TMEM216 protein, starting at the first of the four transmembrane domains and were thus predicted to be null mutations (Fig. 2B). RT-PCR revealed that homozygous knockout animals expressed only mutant mRNA whereas heterozygous animals expressed both wildtype and mutant mRNA (Fig. 2C). Number of homozygous zebrafish for both mutations were expected from Mendelian ratios at one week after fertilization but decreased after two weeks (Table 1). Only one homozygous zebrafish survived to three weeks after fertilization. No homozygous animals were found in ages older than four weeks after fertilization. At the time of sacrifice at 3-dpf, 7-dpf, and 14-dpf, no obvious gross morphologic abnormalities, including edema, pericardial effusion, hydrocephalus, or kidney cysts, were observed in *tmem216^snyR8Δ60^* and *tmem216^snyΔ175^* homozygous zebrafish. The phenotypes observed in the retina of both homozygous knockout animals were indistinguishable. Thus the data from these knockout zebrafish are combined.

**Table 1.** Number of Genotyped Zebrafish

Knockout Lines	3-Dpf	7-Dpf	14-Dpf	21-Dpf	>30-Dpf
*tmem216^snyΔ175^*					
Wildtype		10	37	5	23
Heterozygous		14	60	11	36
Homozygous		9	10	0	0
*tmem216^snyR8Δ60^*					
Wildtype	5	15	63	11	22
Heterozygous	13	27	215	19	49
Homozygous	7	15	37	1	0

### Cone Photoreceptors Were Decreased in TMEM216-Deficient Zebrafish

**Figure 3.**
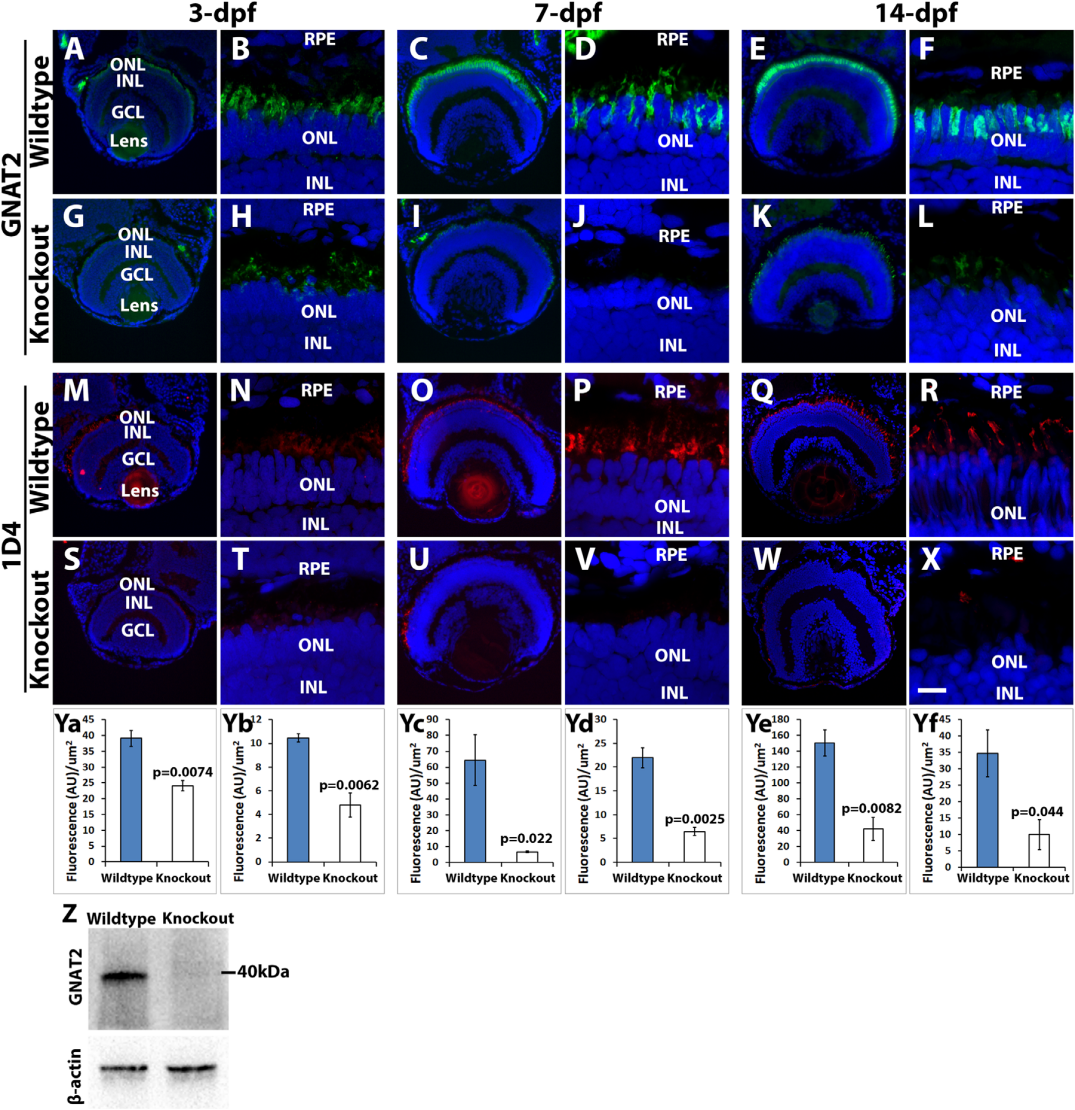
Cone outer segment generation is reduced in *tmem216* knockout fish. Cryosections of whole zebrafish heads were stained with cone OS markers GNAT2 (*green*) and 1D4 (*red*) at 3-, 7- and 14-dpf. All sections were counterstained with DAPI to visualize nuclei. Knockout panels shown were from the *tmem216^snyR8Δ60^* line. Similar results were observed in the *tmem216^snyΔ175^* line. (**A–F**) GNAT2 immunostaining of wild-type retina at 3-, 7-, and 14-dpf, respectively. (**G–L**) GNAT2 staining of *tmem216^snyR8Δ60^* homozygous retina at 3-, 7-, and 14-dpf, respectively. (**M–R**) 1D4 immunostaining of wild-type retina at 3-, 7-, and 14-dpf, respectively. (**S–X**) 1D4 labeling of *tmem216^snyR8Δ60^* homozygous retina at 3-, 7-, and 14-dpf, respectively. (**Ya, Yc, Ye**) Quantification of GNAT2 fluorescence intensity between wildtype and knockout retinas at 3-, 7-, and 14-dpf, respectively. (**Yb, Yd, Yf**) Quantification of 1D4 fluorescence intensity between wildtype and *tmem216^snyR8Δ60^* homozygous retina at 3-, 7- and 14-dpf, respectively. Note that GNAT2 and 1D4 immunoreactivity was significantly reduced at 3-, 7- and 14-dpf (*n* = 3, Student's *t*-test). (Z) Western blotting using anti-GNAT2 was performed on whole head lysates collected from three 7-dpf wildtype and *tmem216^snyR8Δ60^* homozygous zebrafish larvae. The intensity ratio of GNAT2/-β-actin between wildtype and knockout was reduced from 0.88 to 0.27. Scale bar in **X**: 55 µm for **A, G, M**, and **S**; 110 µm for **C, I, E, K, O, U, Q**, and **W**; 6 µm for **B, H, D, J, F, L, N, T, P, V, R,** and **X**. GCL, ganglion cell layer; INL, inner nuclear layer; ONL, outer nuclear layer; RPE, retinal pigment epithelium.

To evaluate the impact of TMEM216 deficiency on cone photoreceptors, we carried out immunofluorescence staining on eye sections from three-, seven-, and 14-days-old zebrafish for anti-GNAT2 (general cone marker, Figs. 3A–3L).^38^ In wildtype zebrafish (Figs. 3A–3F), GNAT2 immunoreactivity was observed at the apical edge of the outer nuclear layer, labeling the outer segments of ultraviolet cones, as well as between the outer nuclear layer and retinal pigment epithelial layer, labeling short-, medium-, and long-wavelength sensitive cone outer segments, in the 3-, 7-, and 14-dpf retina. In *tmem216^snyR8Δ60^* homozygous zebrafish (Figs. 3G–3L), GNAT2 immunoreactivity was reduced at all ages examined. To quantify this effect, we measured intensity of GNAT2 immunofluorescence. GNAT2 immunofluorescence level was reduced in *tmem216^snyR8Δ60^* homozygous zebrafish at 3-, 7-, and 14-dpf (Figs. 3Ya, 3Yc, 3Ye). To further evaluate the loss of GNAT2 protein in *tmem216^snyR8Δ60^* homozygous zebrafish, we performed a GNAT2 Western blot using whole 7-dpf zebrafish larvae head lysate combined from three wild-type and three *tmem216^snyR8Δ60^* homozygous zebrafish. At 7-dpf, the GNAT2/β-actin band intensity ratio was decreased from 0.88 to 0.27 in the knockout (Fig. 3Z). Next, we performed immunostaining with antibody 1D4, a long double cone marker^37^ (Figs. 3M–3X). In wildtype zebrafish (Figs. 3M–3R), 1D4 immunoreactivity was observed in the outer segment layer in 3-, 7-, and 14-dpf throughout the retina. In *tmem216^snyR8Δ60^* homozygous zebrafish (Figs. 3S–3X), 1D4 immunoreactivity was reduced at all ages examined. Like GNAT2, 1D4 immunofluorescence intensity was reduced in *tmem216^snyR8Δ60^* homozygous zebrafish at 3-, 7-, and 14-dpf (Figs. 3Yb, 3Yd, 3Yf). These results indicate that expression of cone photoreceptor-specific GNAT2 and long double cone-specific 1D4 were significantly reduced in TMEM216 knockout zebrafish.

### Mislocalization of GNAT2 and 1D4 Reactivity in Photoreceptors of TMEM216-Deficient Zebrafish

**Figure 4.**
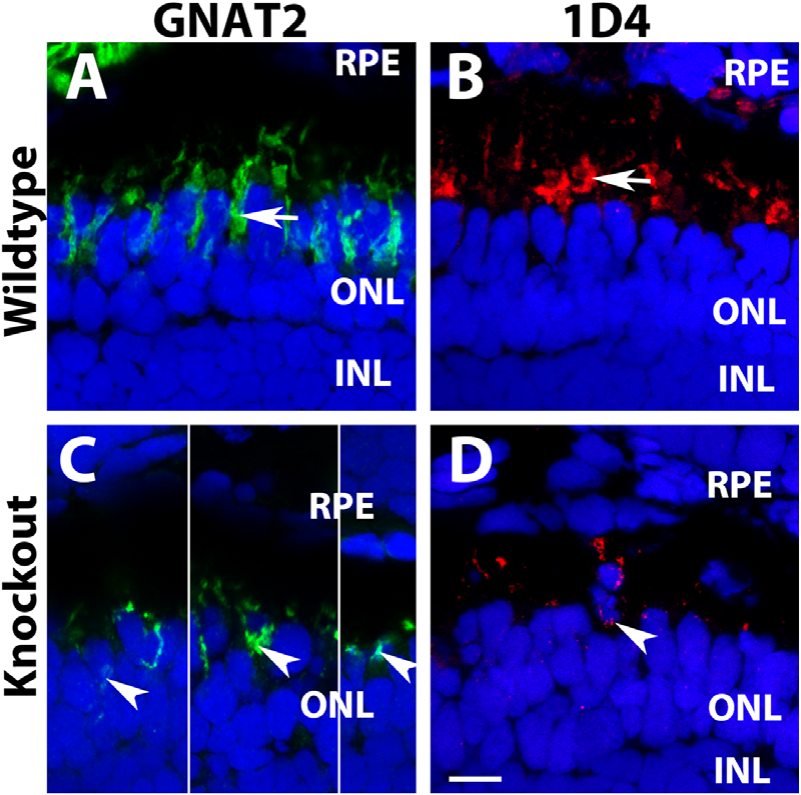
Mislocalization of cone outer segment proteins in *tmem216* knockout photoreceptors. Cryosections were immunostained with GNAT2 (*green*) and 1D4 (*red*). (**A, C**) GNAT2 staining for wildtype and *tmem216^snyR8Δ60^* homozygous retina. Similar phenotypes were observed in the *tmem216^snyΔ175^* homozygous retina. GNAT2 was normally found in the cone outer segments (*arrow* in **A**) in the wildtype retina. Mislocalization of GNAT2 reactivity to the cone cell body was frequently found in the knockout fish (*arrowheads* in **C**). (**B, D**) 1D4 staining of wildtype and knockout retina. 1D4 reactivity was labeling long double cone outer segments in the wildtype (*arrow* in **B**). 1D4 reactivity was frequently found around the cell bodies of the knockout retina (*arrowhead*, in **D**). *Scale bar* in **D**: 6 µm.

GNAT2 protein is highly enriched in cone outer segment with little expression in the inner segment and cell bodies. In light-adapted wildtype animals, GNAT2 is found in the outer segment of cones (Fig. 4A, see arrow for example). In the *tmem216^snyR8Δ60^* homozygous retina, however, GNAT2 immunoreactivity is fragmented, with signal observed in the cell bodies around the nuclei (Fig. 4C, arrowheads). 1D4 antibody recognizes red opsin of long double cones outer segment in zebrafish.^37^ In the wildtype animals, 1D4 immunoreactivity is strongly observed in the outer segment layer (Fig. 4B, arrow). In the *tmem216^snyR8Δ60^* homozygous retina, 1D4 immunoreactivity appeared fragmented and many 1D4 immunoreactive puncta were observed around the cell bodies (Fig. 4D, arrowhead). These results indicated that there is mislocalization of cone outer segment materials to the inner segments and cell bodies of photoreceptors in *tmem216* knockout fish.

### Rod Photoreceptors Were Decreased in TMEM216-Deficient Zebrafish

**Figure 5.**
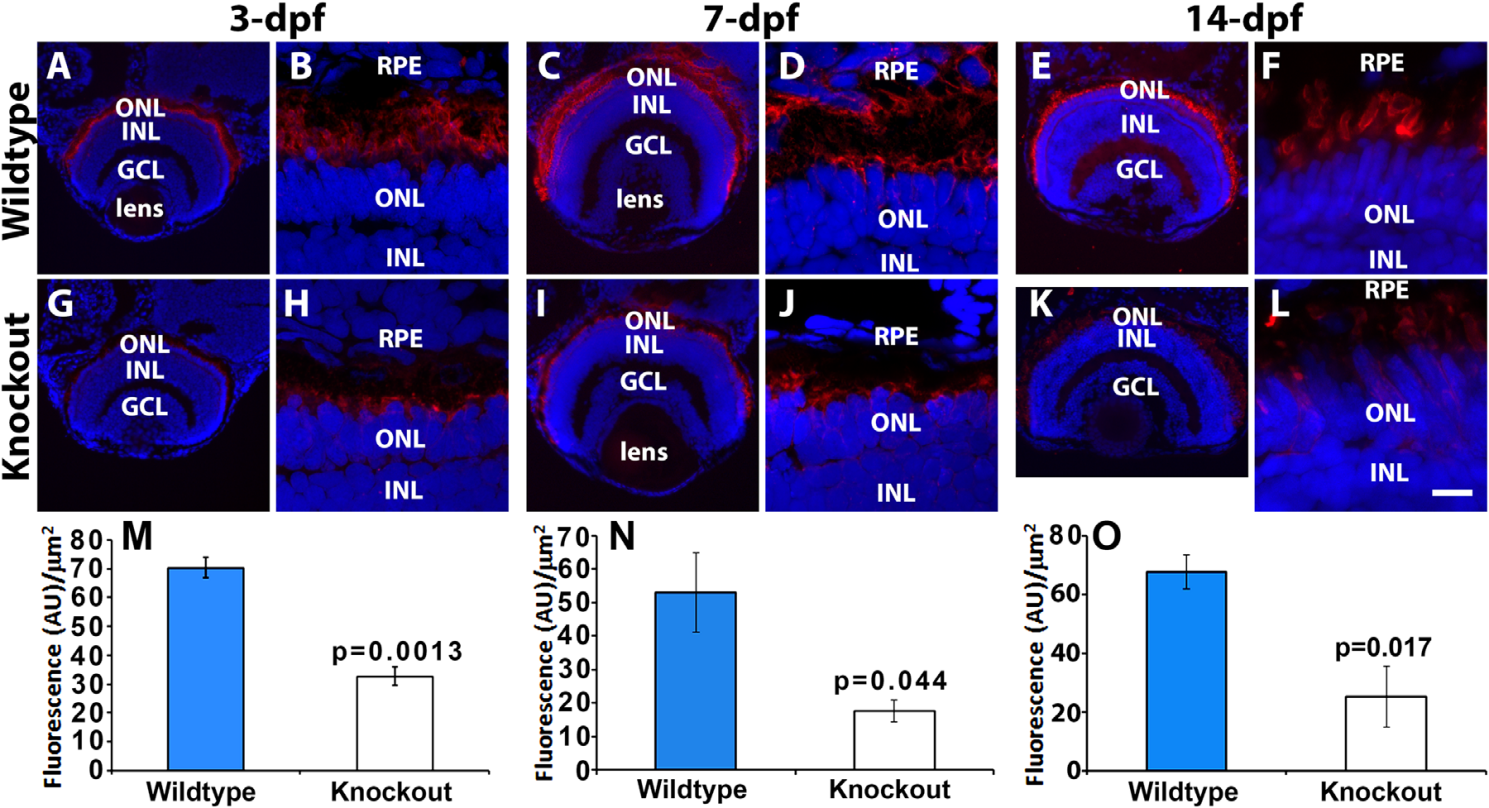
Rod outer segments were reduced in *tmem216* knockout zebrafish. Retinal sections were stained with rhodopsin marker, 4D2 (*red*), and counterstained with DAPI to label nuclei. Knockout images shown represent the *tmem216^snyΔ175^* retina, similar phenotypes were observed in the *tmem216^snyR8Δ60^* homozygous retina. (**A–F**) Wildtype at 3-, 7-, and 14-dpf, respectively. (**G–L**) *tmem216^snyΔ175^* homozygous at 3-, 7-, and 14-dpf, respectively. (**M–O**) 4D2 immunofluorescence intensity was significantly reduced at 3-, 7-, and 14-dpf in *tmem216^snyΔ175^* homozygous retina (*n* = 3, Student's *t*-test). *Scale bar* in **L**: 55 µm for **A** and **G**; 110 µm for **C, I, E,** and **K**; 6 µm for **B, H, D, J, F,** and **L**.

To evaluate rod photoreceptors, we carried out immunofluorescence staining with 4D2 (Abcam) antibody (Fig. 5). In the wildtype animals (Figs. 5A–5F), 4D2 immunoreactivity was observed between the outer nuclear layer and the retinal pigment epithelium throughout the retina. In the *tmem216^snyΔ175^* homozygous animals (Figs. 5G–5L), 4D2 immunoreactivity was sparsely present. Fluorescence intensity of 4D2 reactivity was significantly reduced in *tmem216^snyΔ175^* homozygous zebrafish at three, seven, and 14 days after fertilization when compared with the wildtype animals (Figs. 5M–5O). These results suggested that rod photoreceptors were significantly reduced in *tmem216* knockout zebrafish.

### Mislocalization of 4D2 Immunoreactivity and Disorganization of F-Actin in Photoreceptors of TMEM216-Deficient Zebrafish

**Figure 6.**
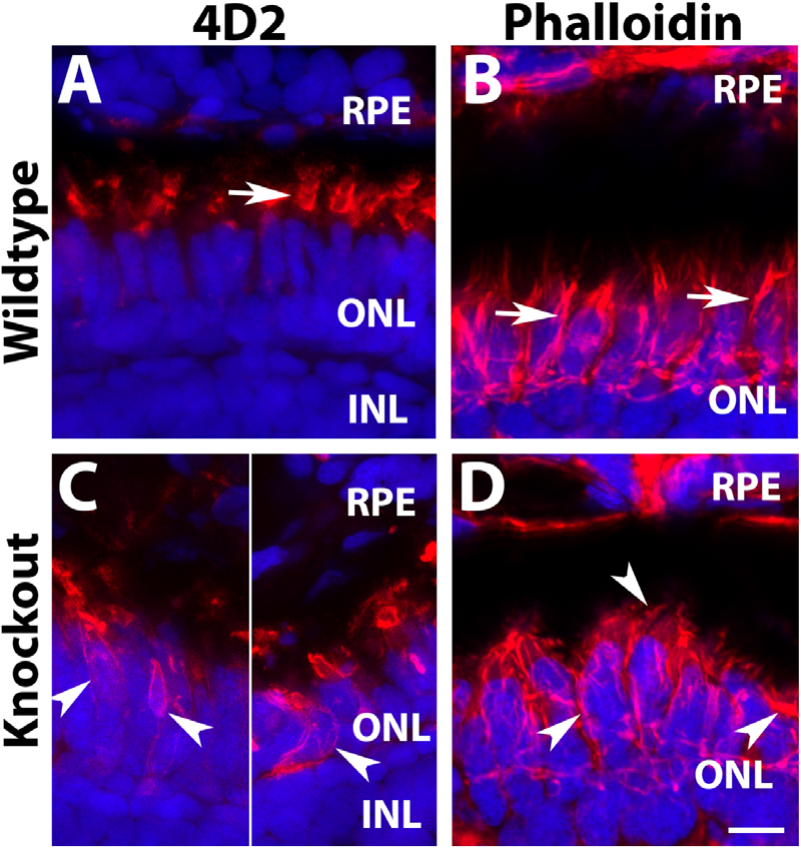
Defective organization of F-actin in *tmem216* knockout photoreceptors. Cryosections were immunostained with 4D2 (*red*). Some sections were stained with Phalloidin-RITC for F-actin (*red*). (**A**, **C**) 4D2 staining of wildtype and *tmem216^snyΔ175^* homozygous retina. 4D2 labels rod outer segments in the wildtype zebrafish (*arrow* in **A**); however, its reactivity was frequently mislocalized to the rod cell body in the knockouts (*arrowheads* in **C**). (**B, D**) Phalloidin staining in wildtype and *tmem216^snyΔ175^* homozygous retina. Vertically oriented F-actin in the wildtype (*arrows* in **B**) were disrupted in *tmem216^snyΔ175^* homozygous retina (*arrowheads* in **D**). *Scale bar* in **H**: 6 µm.

Rhodopsin staining with 4D2 antibody revealed that immunofluorescence activity is highly enriched in the outer segment layer of wildtype zebrafish (Fig. 6A, arrow). In *tmem216^snyΔ175^* homozygous retina, high 4D2 reactivity in the outer nuclear layer was often observed (Fig. 6C, arrowheads). We measured 4D2 immunofluorescence intensity in the outer segment layer and outer nuclear layer and calculated the ratio of fluorescence intensity of cell body layer/outer segment layer. Although the ratio in the wildtype was 0.70 ± 0.0084 (mean ± standard error of the mean [SEM]), the knockout was 1.82 ± 0.288 (mean ± SEM) (*P* = 0.018, Student's *t*-test). These results indicated mislocalization of rhodopsin to the inner segments and cell bodies of photoreceptors in *tmem216* knockout zebrafish.

The actin cytoskeleton is involved in translocation of proteins, such as arrestin, transducin, and cyclic nucleotide-gated channel α-subunit (CNGA1), within photoreceptors. Because several outer segment proteins were found to be mislocalized in the photoreceptors of *tmem216^snyΔ175^* homozygous zebrafish, we used phalloidin-RITC (Sigma-Aldrich) to examine F-actin in *tmem216^snyΔ175^* homozygous zebrafish. In wildtype zebrafish retina, phalloidin staining showed that polymerized F-actin was arranged in a characteristic organization, parallel to the basal-apical radiation axis of photoreceptors (Fig. 6B, arrows). However, in *tmem216^snyΔ175^* homozygous retina, F-actin reactivity was thinner or fragmented with some oriented at an angle relative to the basal-apical axis (Fig. 6D, arrowheads).

### TUNEL-Positive Nuclei Were Increased in TMEM216-Deficient Zebrafish

**Figure 7.**
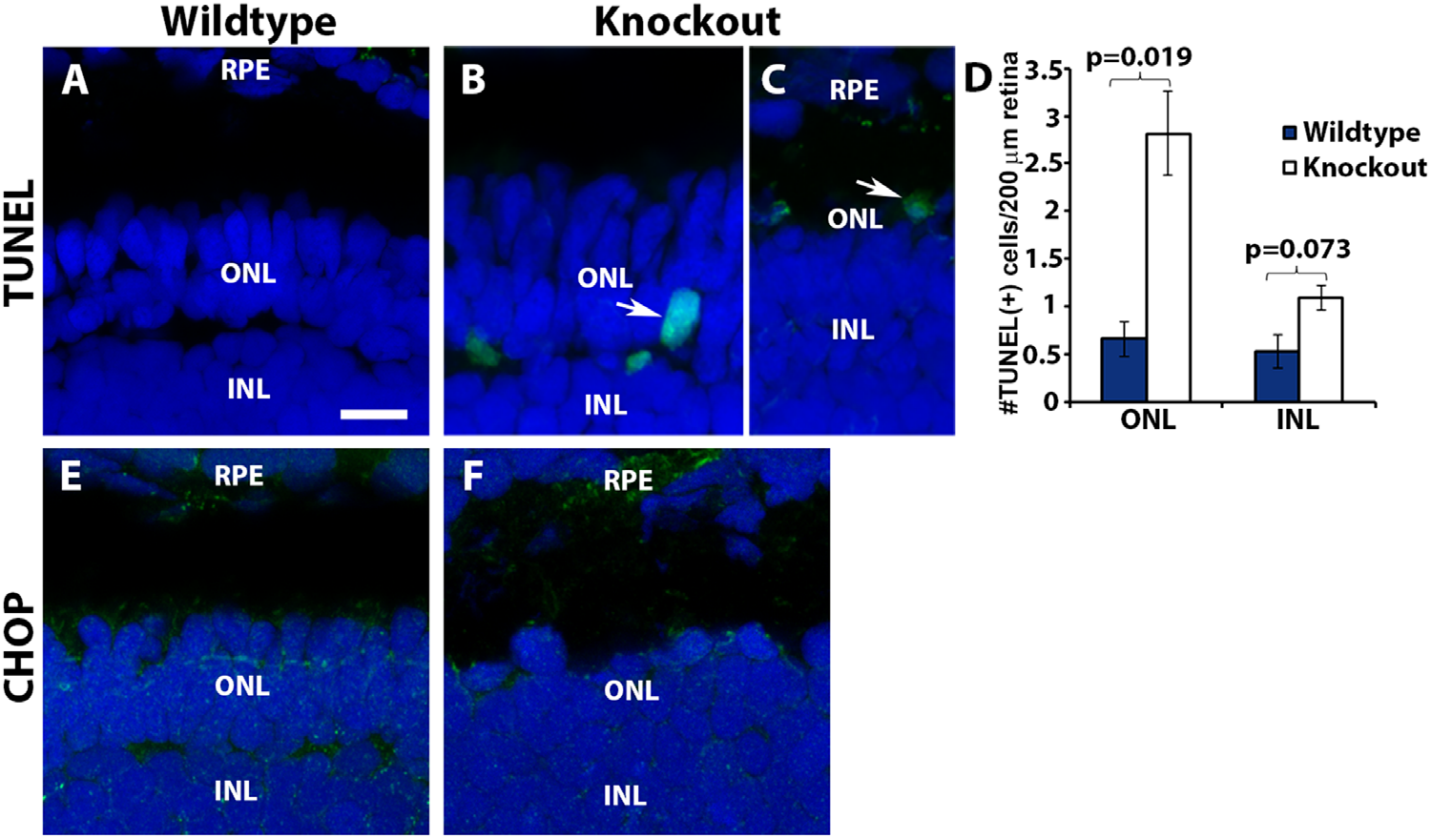
TUNEL-positive photoreceptor nuclei were increased in *tmem216* knockouts. Cryosections from 7-dpf zebrafish were stained by TUNEL assay (*green*) and anti-CHOP (Proteintech), counter stained with DAPI. (**A**) Wildtype. (**B, C**) *tmem216^snyR8Δ60^* homozygous retina. Note TUNEL-positive DAPI-labeled rod (*arrow* in **B**) and cone (*arrow* in **C**) nuclei. (**D**) Quantification of TUNEL-positive nuclei. Number of TUNEL-positive nuclei were significantly increased in the outer nuclear layer of *tmem216^snyR8Δ60^* homozygous animals (*n* = 3, Student's *t*-test); similar phenotype was observed in *tmem216^snyΔ175^* animals. (**E, F**) CHOP staining of wildtype and knockout retina. Immunoreactivity of CHOP antibody was similar between wildtype and *tmem216^snyR8Δ60^* homozygous retina. *Scale bar* in **A**: 6 µm for **A, E**, and **F**; 8 µm for **B** and **C**.

To determine photoreceptors loss by programmed cell death, we carried out terminal deoxynucleotidyl transferase dUTP nick end labeling (TUNEL) assay as described.^38^ TUNEL-positive nuclei in the outer nuclear layer of *tmem216^snyR8Δ60^* homozygous zebrafish were frequently observed (arrows, Figs. 7B and 7C) and significantly increased over the wildtype animals (Fig. 7D). These results indicate that apoptotic cell death in photoreceptors were increased in *tmem216* knockout zebrafish.

The *tmem216* mutation in zebrafish may cause ER stress, which might result in photoreceptor cell death. To evaluate this, we stained 7-dpf wildtype and knockout retina with anti-CCAAT/-enhancer-binding protein homologous protein (CHOP). In the wildtype and *tmem216^snyR8Δ60^* homozygous zebrafish, CHOP labeling was present throughout all layers of the neural retina, including the photoreceptor inner segments and cell bodies (Figs. 7E and 7F). However, we did not observe an increase in CHOP expression in the knockout retina, suggesting a lack of significant ER stress in TMEM216-deficient photoreceptors.

### Photoreceptor Axonemes Were Shorter in TMEM216-Deficient Zebrafish

**Figure 8.**
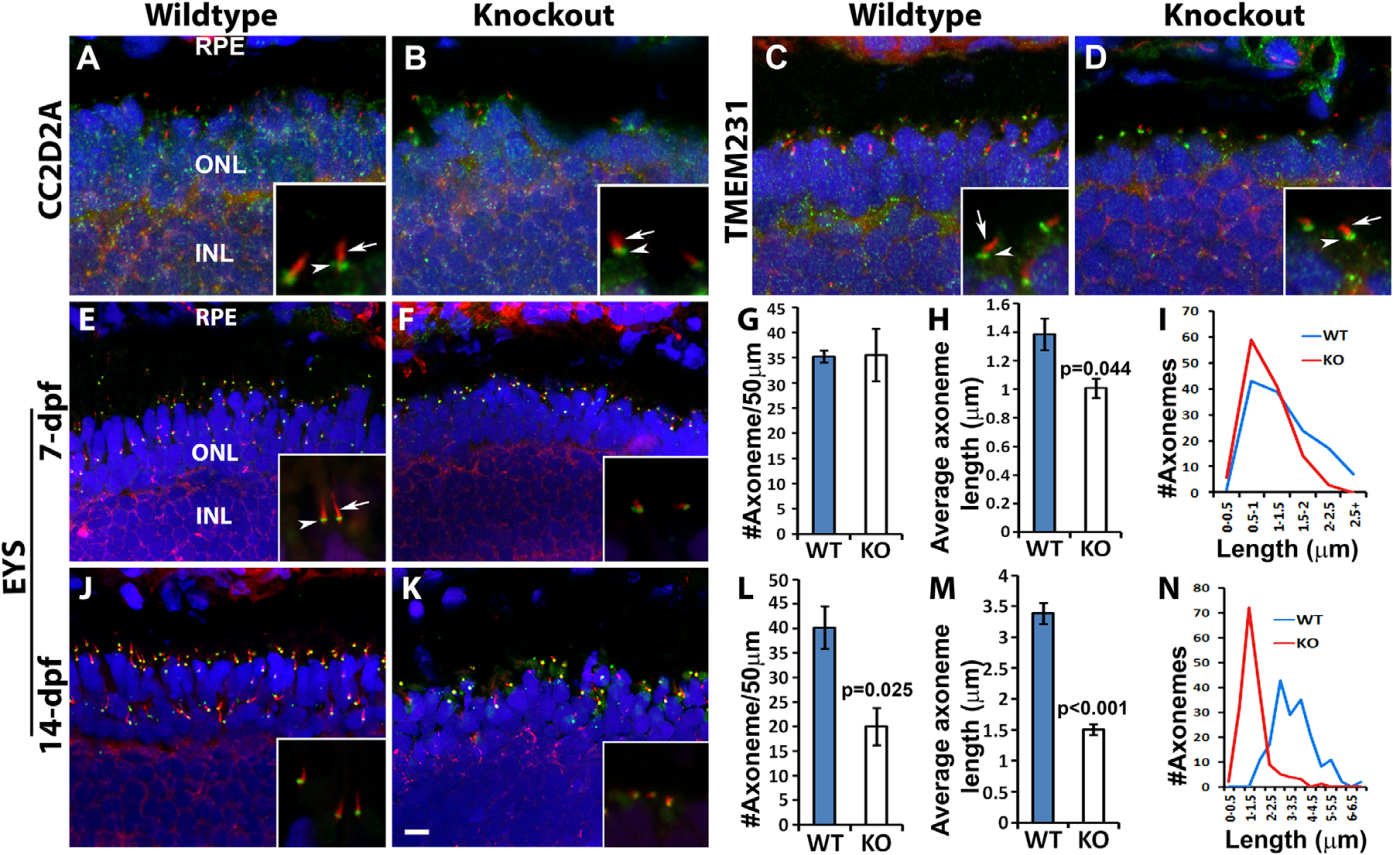
Length of photoreceptor axonemes was reduced in knockout zebrafish. Cryosections were immunostained with axoneme marker, acetylated α-tubulin (*red*), and connecting ciliary region markers CC2D2A, TMEM231, and EYS (*green*). Sections were then counterstained with DAPI to visualize nuclei. (**A, B**) Acetylated α-tubulin (*red, arrow*) and CC2D2A (*green, arrowhead*) double immuno-labeling of 7dpf wildtype and *tmem216^snyΔ175^* homozygous retina. (**C, D**) Acetylated α-tubulin (*red, arrow*) and TMEM231 (*green, arrowhead*) double immuno-labeling of 7dpf wildtype and *tmem216^snyΔ175^* homozygous retina. (**E, F**) Acetylated α-tubulin (*red, arrow*) and EYS (*green, arrowhead*) double immuno-labeling of 7dpf wildtype and *tmem216^snyΔ175^* homozygous retina. Note the reduction in axoneme length in the knockout zebrafish (**F, inset**). (**J, K**) Acetylated α-tubulin and EYS double immuno-labeling of 14-dpf wildtype and *tmem216^snyΔ175^* homozygous retina. (**G–I**) Quantification of 7-dpf axoneme number and length. Note the reduction in average axoneme length (*n* = 3, Student's *t*-test) and the shift of axoneme lengths toward lower length bins in the knockout. (**L–N**) Quantification of 14-dpf axoneme number and length (*n* = 3, Student's *t*-test). *Scale bar* in **K**: 6 µm for **A–F**, **J,** and **K**; 1.5 µm for insets.

TMEM216 is a member of the tectonic complex highly enriched at the transition zone of primary cilia.^26^^,^^27^^,^^39^ Tctn1, a member of this complex, is required for ciliogenesis of some but not all cell types. We therefore evaluated whether TMEM216 deletion affected localization of other transition zone proteins, CC2D2A (Figs. 8A and 8B) and TMEM231 (Figs. 8C and 8D), by immunofluorescence staining of 7-dpf zebrafish. In the wildtype animals, CC2D2A and TMEM231 immunoreactivity (green fluorescence) was localized to the basal end of the acetylated α-tubulin (Figs. 8A and 8C, arrowheads), an axoneme marker. Expression of both CC2D2A and TMEM231 at the basal end of acetylated α-tubulin was not affected in *tmem216^snyΔ175^*homozygous animals (arrowheads in Figs. 8B and 8D), indicating that CC2D2A and TMEM231 was not affected by TMEM216 deletion. Similarly, localization of EYS, an extracellular matrix protein expressed near the connecting cilia was not affected by TMEM216 deletion (green fluorescence, Figs. 8E–8F, 8J, 8K).

With regard to acetylated α-tubulin-labeled axonemes, they were observed throughout the outer nuclear layer and outer segment layer at 7-dpf in the wildtype retina (Fig. 8E). In *tmem216^snyΔ175^* homozygous zebrafish, axonemes were also observed throughout these layers at 7-dpf (Fig. 8F) with similar numbers as the wildtype (Fig. 8G). However, the length of acetylated α-tubulin-positive ciliary axoneme was reduced in *tmem216^snyΔ175^* homozygous zebrafish retina at 7-dpf (Figs. 8F, 8H, 8I). These results indicated that TMEM216 knockout photoreceptors generate cilia, but the length of photoreceptor cilia was shorter than wildtype animals. At 14-dpf, density of acetylated α-tubulin in *tmem216^snyΔ175^* homozygous zebrafish was reduced when compared to the wildtype retina (Figs. 8K, 8L). Similar to the 7-dpf zebrafish knockout axonemes, the average axoneme length in the 14-dpf *tmem216^snyΔ175^* homozygous zebrafish was shorter than the wildtype (Figs. 8K, 8M, 8N). Reduced number of acetylated α-tubulin in *tmem216* knockout retina at 14-dpf was expected as a result of photoreceptor degeneration. These results indicate that photoreceptor primary cilia were formed in *tmem216* knockout retina but were shortened compared with the wildtype.

### TMEM216 Is Required for Normal Genesis of Outer Segment

**Figure 9.**
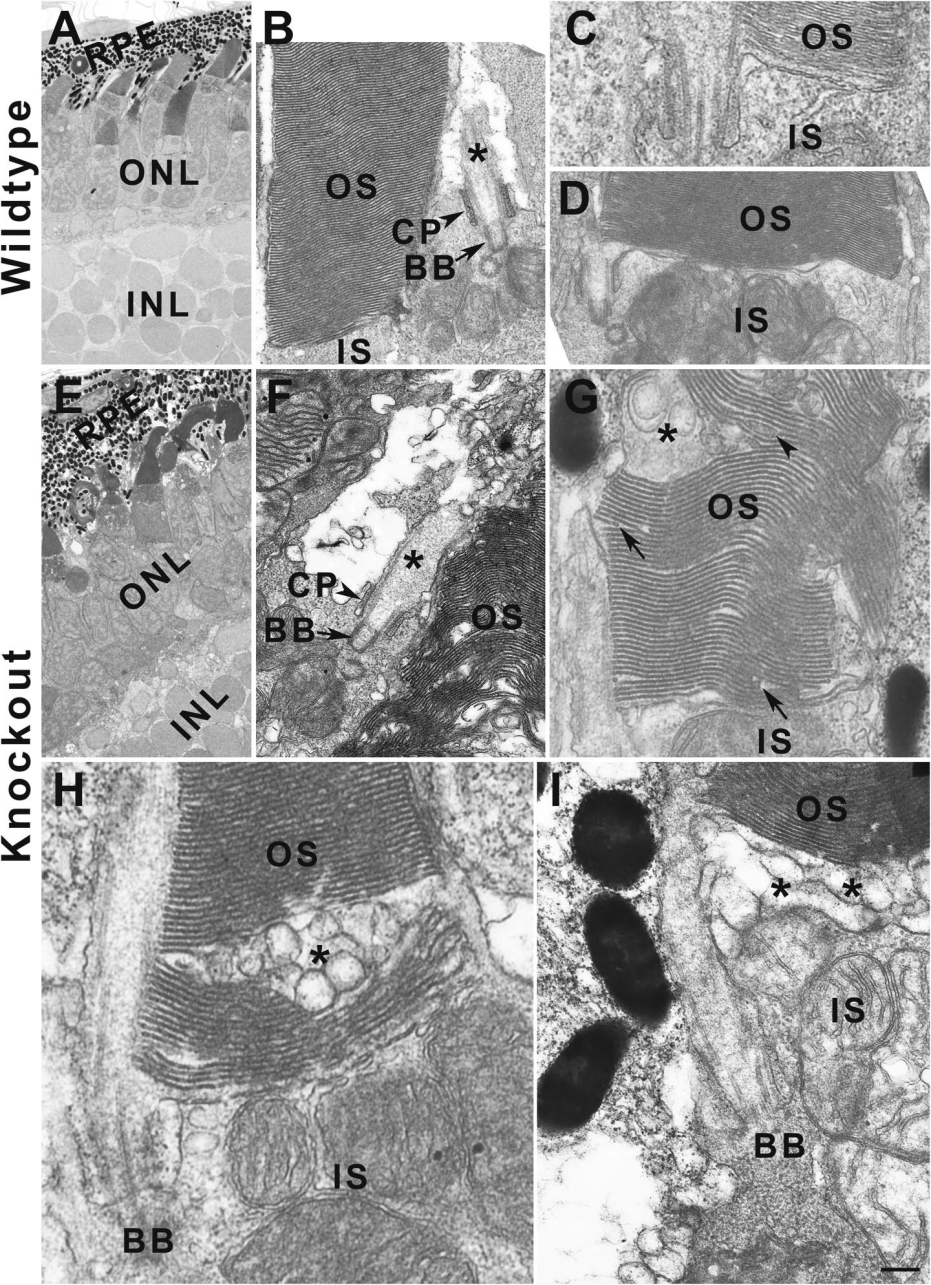
*tmem216* knockouts exhibit abnormal outer segment disc morphology. Eyes from 7-dpf wildtype and *tmem216^snyR8Δ60^* zebrafish were processed for transmission EM. (**A–D**) Wildtype retina. Note that the outer segments with uniform discs. (**E–I**) *tmem216^snyR8Δ60^* homozygous retina. Although photoreceptors in the *tmem216^snyR8Δ60^* homozygous retina elaborated cilia (*asterisk* in **F**), the outer segments of these photoreceptors exhibit multiple defects. Abnormalities manifested as large vacuoles within the outer segment (*asterisks* in **G** and **H**), large vacuoles at the base of the outer segment (*asterisks* in **I**), shortened discs (*arrows* in **G**), abnormal disc morphology (*arrowhead* in **G**). *Scale bar* in **I**: 4 µm for **A** and **E**; 400 nm for **B, D,** and **F**; 200 nm for **C, G, H,** and **I**. BB, basal body; CP, ciliary pocket; IS, inner segment; OS, outer segment.

To determine the impact of TMEM216 deletion on the ultrastructure of photoreceptors, we carried out transmission electron microcopy analysis of *tmem216^snyR8Δ60^* homozygous zebrafish at 7-dpf. Photoreceptors with elaborated outer segments were observed for both rods and cones in wildtype and *tmem216^snyR8Δ60^* homozygous zebrafish (Figs. 9A, 9E). Since acetylated α-tubulin staining showed similar pattern of immunoreactivity at 7-dpf in the knockout retina, it is expected that ciliogenesis of photoreceptors was not affected at the ultrastructural level. Indeed, newly elaborated cilia with clear basal bodies (arrows), ciliary pockets (arrowheads), and axonemes (asterisks) in *tmem216^snyR8Δ60^* homozygous retina (Fig. 9F) were phenotypically similar to the wildtype retina (Fig. 9B). However, multiple abnormalities were observed in the outer segment in *tmem216^snyR8Δ60^* homozygous animals. In the wildtype retina, outer segment of rods (Figs. 9B, 9C) and cones (Fig. 9D) with uniform discs were observed at 7-dpf. However, in *tmem216^snyR8Δ60^* homozygous photoreceptors, some outer segments exhibit scrambled disc structures (Fig. 9F), with large vacuoles within the outer segment (asterisks in Figs. 9G, 9H), vacuoles near the base of outer segment/apical end of inner segment (asterisks, Fig. 9I), and uneven disc sizes with apparent shortened discs that do not span the width of the photoreceptor outer segment (arrows, Fig. 9G). We counted outer segment exhibiting normal disc morphology, shortened discs, and presence of vacuoles from images focused on photoreceptors showing the connecting cilia (Table 2) from 3 wild-type and 3 *tmem216^snyR8Δ60^* homozygous zebrafish. The majority of the outer segment in TMEM216 knockout photoreceptors exhibited outer segment disc defects (*P* < 0.0001, Fisher's exact test). These data indicate that loss of TMEM216 does not affect photoreceptor ciliogenesis but, rather, outer segment genesis.

**Table 2.** Quantification of Outer Segment Defects

Outer Segment Morphology	Wild-Type	TMEM216 Knockout
Normal	39	8
Shortened discs	2	10
Vacuoles	1	8
Shortened discs and vacuoles	0	5

*P* = 3.79 × 10^−9^, Fisher's exact test.

## Discussion

We deleted TMEM216 in zebrafish with CRISPR-mediated genome editing. Homozygous *tmem216* knockout animals died before three weeks after fertilization. Although photoreceptors were generated in TMEM216-deficient animals and elaborated cilia, the ciliary length was shortened. Loss of photoreceptors was observed at 3-, 7- and 14-dpf. This was accompanied by mislocalization of outer segment proteins including GNAT2, cone opsin, and rhodopsin. F-actin morphology was altered in the knockout photoreceptors. EM analysis revealed that *tmem216* knockout photoreceptors elaborated outer segment with ultrastructural morphological defects including disorganized discs, presence of large vacuoles within the outer segment or at the base of outer segment, un-uniform disc sizes. These results indicate that TMEM216 is required for normal outer segment disc morphogenesis and photoreceptor survival in zebrafish.

The transition zone Tectonic/B9D1 complex proteins play critical roles in ciliary trafficking and genesis.^26^^,^^27^^,^^37^ Tectonic complex protein member TCTN1 is required for cilia formation in select types of cells such as those in the node and neural tube cells but not in the limb bud and perineural mesenchyme.^23^ As a member of the Tectonic/B9D1 complex proteins, TMEM216 is also involved in ciliogenesis. *Tmem216* mutations in patient fibroblasts result in impaired ciliogenesis and centrosomal docking.^12^ Morpholino-mediated knockdown of *tmem216* in zebrafish reveal ciliopathy-associated phenotypes, such as curved/kinked tail, pericardial effusion and gastrulation defects.^12^^,^^25^ The effect of morpholino knockdown of *tmem216* on the zebrafish retina was not reported. Here, we show that loss of TMEM216 does not affect localization of tectonic complex proteins CC2D2A and TMEM231. The *tmem216* knockout zebrafish exhibit normal number of photoreceptor axonemes at seven days after fertilization, indicating that a normal number of photoreceptor cilia were generated. However, the axoneme length was reduced in *tmem216* knockout zebrafish. Immunostaining for rod and cone outer segment markers showed significantly reduced reactivity in *tmem216* knockout retinas. These results indicated that TMEM216 was not absolutely required for elaboration of the connecting cilia in photoreceptors, though these cilia were shortened.

Mutations in tectonic complex genes often cause severe retinal phenotypes. Besides cystic kidney and cerebellar vermis hypoplasia,^40^^,^^41^ *Ahi1-*null mice lack photoreceptor outer segments and are reported to have severe retinal degeneration at early ages.^42^^,^^43^ Loss of *TCTN2* or *TCTN3* results in microphthalmia in mice.^44^^,^^45^ A frameshift mutation in *Cep290* in the rdAc Abyssinian cat has been reported to result in rod-cone dystrophy, with complete blindness occurring between 3-5 years of age.^46^^–^^49^ EM studies of the rdAc cat revealed accumulation of vesicles near the rod connecting cilium.^49^ Similar to the rdAc cat, loss of *Cep290* in mice, results in loss of photoreceptor outer segments as well as a shortening of photoreceptor inner segments.^31^ *Tmem67/mks3* is crucial in photoreceptor health, because *tmem67*-null rat photoreceptors do not develop outer segments.^50^ CC2D2A mutant zebrafish exhibits disruptions of rod and cone outer segments.^21^^,^^43^ Photoreceptors in CC2D2A-deficient zebrafish can grow cilia but outer segment genesis is defective, with accumulation of vesicles in the photoreceptor inner segments.^43^ In *tmem216* knockout zebrafish, photoreceptor outer segment exhibited multiple defects in disc morphology, with the presence of vacuoles throughout the photoreceptor outer segment, as well as shortened outer segment discs. Together with mislocalization of outer segment protein in *tmem216* knockout photoreceptors, these data indicated that TMEM216 plays critical roles in photoreceptor disc morphogenesis and extension, supporting an essential role of Tectonic/B9 complex proteins in morphogenesis of the outer segment.
